# Comparison of a non-invasive point-of-care measurement of anemia to conventionally used HemoCue devices in Gambella refugee camp, Ethiopia, 2022

**DOI:** 10.1371/journal.pone.0313319

**Published:** 2025-01-13

**Authors:** Frederica Smith, Meseret Woldeyohannes, Millicent Lusigi, Kaitlyn L. I. Samson, Blessing Tapera Mureverwi, Dorothy Gazarwa, Naser Mohmand, Terry Theuri, Eva Leidman

**Affiliations:** 1 US Centers for Disease Control and Prevention, Global Health Center, Atlanta, GA, United States of America; 2 Ethiopian Public Health Institute (EPHI), Food Science and Nutrition Research Directorate, Addis Ababa, Ethiopia; 3 United Nations High Commissioner for Refugees, UNHCR, Addis Ababa, Ethiopia; 4 Action Against Hunger, AAH, Toronto, Canada; 5 United Nations High Commissioner for Refugees, UNHCR, Nairobi, Kenya; 6 United Nations High Commissioner for Refugees, UNHCR, Geneva, Switzerland; 7 US Centers for Disease Control and Prevention, National Center for Immunization and Respiratory Diseases, Atlanta, GA, United States of America; Shoklo Malaria Research Unit, THAILAND

## Abstract

Annual surveys of refugees in Gambella, Ethiopia suggest that anemia is a persistent public health problem among non-pregnant women of reproductive age (NP-WRA, 15–49 years). Measurement of anemia in most refugee camp settings is conducted using an invasive HemoCue 301. We assessed the accuracy and precision of a non-invasive, pulse CO-oximeter in measuring anemia among NP-WRA in four Gambella refugee camps. We conducted a population-representative household survey between November 7 and December 4, 2022. Hemoglobin (Hb) concentration was measured by HemoCue 301, using capillary blood, and Rad-67, a novel non-invasive device. We collected four measurements per participant: two per device. We calculated Rad-67 bias and precision of Hb measurements and sensitivity and specificity of detecting anemia. Of the 812 NP-WRAs selected, 807 (99%) participated in the study. Anemia was detected in 39% of NP-WRA as classified by the Rad-67 compared with 47% of NP-WRA as classified by the HemoCue 301. Average bias of Rad-67 measurements was 1.1 ± 1.0 SD g/dL, using HemoCue 301 as a comparator. Absolute mean difference between the first and second measurements was 0.9 g/dL (95% CI 0.8, 0.9) using the Rad-67, compared with 0.6 g/dL (95% CI 0.5, 0.6) using the HemoCue 301. The Rad-67 had 49% sensitivity and 70% specificity for detecting anemia, compared with the HemoCue 301. The Rad-67 can be a useful tool for anemia screening; however, lower accuracy and precision, and poor sensitivity suggest it cannot immediately replace the HemoCue 301 in the study area.

## Introduction

Anemia is a significant public health concern. Globally, in 2019, the estimated prevalence of anemia was 30% among women of reproductive age (WRA, 15–49 years) and 40% among children aged 6–59 months [1]. In a 2022 nutrition survey of refugees living in six camps in Gambella, Ethiopia, anemia prevalence had a range of 27.8–56.4% among WRA and 44.6–68.8% among children aged 6–59 months, thus classified as a severe public health problem [2]. The causes of anemia are complex, including modifiable risk factors such as micronutrient deficiencies and infection, and can lead to increased morbidity and mortality [3]. The World Health Organization (WHO) released an accelerated anemia control framework, recognizing the complexity of anemia etiology and the slow advancement towards global targets [4]. A review of the United Nations Refugee Agency’s (UNHCR) Anemia Strategy [5] highlighted persistently high levels of anemia in refugee camps despite programming efforts [6]. Among refugees, the degree of anemia may be worsened by inadequate nutrient intake or increased exposure to infectious agents [7,8]. Nonetheless, anemia prevalence estimates among displaced women and children are similar to global estimates [9].

The effective prevention, treatment, and monitoring of anemia are dependent on the availability of appropriate diagnostic tools. In field settings, including among populations in displacement camps, portable photometric point-of-care (POC) analyzers (e.g., HemoCue® devices) represent the current standard of care for anemia diagnosis [10,11]. This method, however, poses challenges as it requires the collection of a capillary blood sample (~10 μL) and thus the regular procurement of consumable supplies for sample collection [10], which may not be easily obtained in remote field settings. Ultimately, there is a need for easy-to-use, non-invasive tools with limited marginal costs to diagnose anemia in such settings. One potential solution is the use of pulse CO-oximeter devices, which measure oxygen saturation, pulse rate, perfusion index, and total hemoglobin (Hb) via Hb-bound oxygen and carbon monoxide levels in an individual–eliminating the need for a blood sample [10].

Numerous studies in high-income and healthcare settings have compared pulse CO-oximeter devices with both HemoCue devices and automated hematology analyzers (a laboratory-based test). Pooled data from a meta-analysis of 39 studies found that, when compared with automated hematology analyzers, the HemoCue 201+ had a mean difference of 0.10 g/dL [95% prediction interval (PI): -0.14, 0.33] while Masimo Pronto-7 and Radical-7 pulse CO-oximeter devices had mean differences of 0.18 g/dL (95% PI: -0.35, 0.70) and -0.11 g/dL (95% PI: -0.42, 0.20) [12], supporting the potential use of pulse CO-oximeters in humanitarian settings. A more recent review found that the HemoCue 301, a version commonly used in the field because of its stability at environmental extremes of temperature and humidity, produced higher Hb concentrations (0.05–0.61 g/dL) when compared with a laboratory reference; however, values were within an acceptable accuracy threshold [10]. Results for mean Hb concentrations from the Masimo Radical-7 and Pronto-7 devices, in comparison with a laboratory reference, displayed more variation but were within the acceptable ±7% threshold for mean concentration variation [10]. The Rad-67™ Pulse CO-Oximeter® (Rad-67) is a new version of the Masimo Radical-7 that provides spot-check monitoring of Hb concentration and is indicated for use in non-pregnant adults without renal disease.

Experts have highlighted the need to determine the most reliable measure of Hb in population and field-based surveys [13]. While pulse CO-oximeter devices have been extensively tested, data primarily come from high-income and clinical settings. A Japanese study in children with stable disease (with few exclusions) aged 1–5 years old that compared Hb concentration measured using the Rad-67 to a laboratory reference found a mean bias of 0.188 ± 0.919 g/dL, with a difference within 1.0 g/dL in 75% of patients [14]. A highly significant correlation (*r* = 0.812, p <0.01) between the Rad-67 and laboratory reference and similar bias (0.146 ± 1.39 g/dL) were observed in a study of patients who visited the emergency department and required bloodwork at a hospital in Jordan [15]. Further research is needed to establish whether these validity findings can be replicated in humanitarian and field settings, which are less controlled than a clinical setting and subject to extreme temperatures and humidity. In these settings, the durability of the device and its performance are key considerations.

This study was motivated by UNHCR, the Government of Ethiopia Refugee and Returnee Services, and non-governmental organizations interest in a potential alternative to invasive Hb measurement in Ethiopia’s refugee camps. Evidence supports the potential financial, public health surveillance, and programmatic benefits of the Rad-67. This comparison of devices aimed to determine the accuracy, precision, and user experience of a non-invasive pulse CO-oximeter (the Rad-67) for measuring anemia among non-pregnant WRA (NP-WRA) living in camps in Gambella Region, Ethiopia during a representative community-based nutrition survey.

## Methods

### Study design and setting

This study of non-invasive (pulse CO-oximetry), point-of-care measurement of anemia was carried out in four of seven refugee camps within Gambella Region, Ethiopia. The region hosts an estimated population of 305,677 refugees, the majority of whom are of South Sudanese origin [16].

Data collection was nested within the routine UNHCR Standardized Expanded Nutrition Survey (SENS) [17] for refugee populations. The annual SENS measures the population prevalence of anemia using a HemoCue 301 to measure Hb in capillary blood. In addition to routine measurements in the SENS, this study measured Hb using a non-invasive Rad-67 [18].

### Sample size

Using a two-sample paired-means test, a total sample size of 271 NP-WRA for a pooled analysis (across all camps) was sufficient to detect a 0.2 g/dL difference in Hb concentration between the HemoCue 301 and Rad-67 with 90% power, alpha of 0.05, assumed correlation of 0.8, and a standard deviation of Hb distribution from previous studies of 1.6 g/dL [19]. The minimum precision of both HemoCue 301 and Rad-67 devices is 0.1g/dL [18]. Accounting for a 10% test failure or refusal rate, a sample of at least 300 NP-WRA was necessary for the study.

### Data collection

A cross-sectional study of a non-invasive anemia measurement was conducted within the routine UNHCR SENS in Jewi, Kule, Nguennyiel, and Pinyudo I camps between November 7 and December 4, 2022. Eligible participants were self-reported NP-WRA residing in households who were generally healthy (by self-report), registered as refugees, and resided in one of the Gambella Refugee Camps. Individuals with self-reported renal disease, apnea, current pregnancy, or with unknown pregnancy status were excluded from the survey. Households were randomly sampled from a UNHCR list of refugee households without replacement.

Surveys were administered by trained health professional teams composed of a team leader, interviewer, translator, lead anthropometric measurer, anthropometric assistant, and Hb measurer. Teams were trained for five days, including standard SENS training, Rad-67 device training, a day for standardization using the Rad-67, and pilot testing of the survey and instrument.

Surveys were written in English, formatted in KoBoCollect v2021.2.4 (survey data collection and management tool), and administered in English, with verbal translation into Nuer or Agnuak, based on responder preference. Data were collected using password-protected Lenovo tablets, stored securely, and reviewed daily to assess quality and completeness. No personal identifiers were collected.

Four Hb measurements were taken from each participant following the standard procedures [18,20]. First, non-invasive Hb concentration and perfusion index measurements were taken from the middle and ring finger on the non-dominant hand using the Rad-67. Sensors, approved for measurement of total hemoglobin in pediatric patients weighing 10–50 kg and adult patients weighing more than 30 kg, were used to take Rad-67 measurements. Weight, in kilograms, was measured by seca scales to test if these sensors would work well for adults with heavier weights. Second, a capillary blood sample was taken from the ring finger on the non-dominant hand, placed directly into the microcuvette, and tested using the HemoCue 301. A single finger prick was conducted, and the third and fourth drops of blood were tested. Due to supply limitations of commodities, second HemoCue 301 measurements were not obtained for participants in the Pinyudo I camp. Blood samples were collected by experienced health professionals or laboratory technicians.

Temperature and humidity were recorded at the time of Hb measurements or once per day (in zones with limited cellular signal) to evaluate whether the precision of Hb measurements was impacted at extreme temperature and humidity readings. Temperature and humidity were not recorded for Pinyudo I camp due to poor connectivity.

### Data analysis

Mean and standard deviation of weight and perfusion index of NP-WRA, temperature, and humidity were calculated for each camp. Age and breastfeeding status of NP-WRA were summarized for each camp.

To determine the accuracy of measuring Hb, differences between Hb concentrations measured by both HemoCue 301 and Rad-67 were evaluated. Use of an automated hemoglobin analyzer as a gold standard was not feasible in this setting, given equipment availability in regional labs. As such, differences in estimated Hb using the two point-of-care devices were evaluated. For simplicity, this difference is referred to as ‘accuracy’; doing so does not suggest the HemoCue 301 as a gold standard for Hb concentration measurement. Bias was defined as the absolute difference between Hb concentrations obtained from the Rad-67 and HemoCue 301. Mean bias, standard deviation, 95% confidence intervals, 95% limits of agreement (LOA), and technical error of measurement (TEM) were calculated. The LOA was calculated as the 95% precision interval for individual differences, and the TEM was calculated as the square root of measurement error variance [21,22]. Bland-Altman plots were created to visually assess accuracy as the correlation between bias (y-axis) and mean Rad-67 and HemoCue 301 measurements (x-axis). A Pearson’s correlation coefficient was calculated to determine the agreement between both devices. Implausible values (defined as Hb <3g/dL) were excluded from the analysis.

To determine the precision of measuring anemia, differences between first and second Hb concentrations measured by each of the two point-of-care devices, the HemoCue 301 and Rad-67, were independently evaluated. Precision was defined as the closeness of repeat measurements from the same device. Mean difference, 95% confidence interval, TEM, and 95% LOA were calculated. A Pearson’s correlation coefficient was calculated to determine agreement between repeat measurements. Values beyond the devices’ measurement ranges (0.0–25.0 g/dL for the Rad-67 and 0–25.6 for the HemoCue 301) were excluded.

The Gambella region experiences extreme temperature and heat conditions above the manufacturer’s specifications for operating temperature (0–35°C) [18]. Thus, univariate linear regressions were performed to determine if temperature and humidity influenced Rad-67 precision and accuracy and other characteristics hypothesized to be potentially associated informed by prior research, including women’s weight, age, perfusion index, and location (camp). Considering the correlation between temperature and humidity, multivariate linear regressions were conducted between temperature and humidity and each of these variables.

Anemia classification was determined per World Health Organization (WHO) guidelines [23]. Prevalence of total (Hb concentration <12.0 g/dL), mild (Hb concentration 11.0–11.9 g/dL), moderate (Hb concentration 8.0–10.9 g/dL), and severe (Hb concentration <8.0 g/dL) anemia were calculated using only the first measurement and using the mean of the individual two Hb concentrations measured by the HemoCue 301 as the standard. Sensitivity, specificity, and correct classification rates for total, mild, moderate, and severe anemia were calculated, as measured by the Rad-67 using HemoCue 301 as a comparator. All statistical analyses were conducted in R Studio Statistical Software (version 1.4.1717; R Foundation for Statistical Computing). A p-value <0.05 was considered statistically significant.

### Focus group discussions

Focus group discussions were conducted with survey enumerators and supervisors to capture their experiences using the devices (**S1 File**). Discussions were held after data collection was completed and facilitated using focus group discussion guides exploring the experiences using the pulse CO-oximeter devices. Eligible participants were adults (over 18 years of age) who participated as survey enumerators and supervisors. Focus groups were led by the research investigator (acting as moderator), a notetaker, and a logistics coordinator. Two focus groups per camp were organized and included 15–23 survey enumerators and supervisors.

Focus groups were conducted in Amharic, recorded, transcribed, and translated into English. Detailed notes were included. Data were organized by discussion questions and synthesized in Microsoft Excel. Responses were categorized using a deductive coding process according to pre-identified areas of interest: experience training on and using the devices, perception and acceptability of women using the devices, barriers to measurement, and considerations for future use. Data were reviewed within these categories, and an initial set of codes were created. Responses were then re-categorized to include new themes that emerged from the coding process. Qualitative data were used to inform the quantitative results.

### Ethics approval

The Ethiopian Public Health Institute Institutional Review Board approved the study (protocol number: EPHI-IRB-401-2022). This project was deemed non-exempt human subjects research by the CDC with reliance on EPHI determination as the IRB of record and conducted consistent with applicable federal law and CDC policy. The Government of Ethiopia Refugee and Returnee Services granted permission to conduct the work in the Gambella refugee camps. Written informed consent was obtained directly from eligible women 18–49 years and from a parent or caregiver of all eligible women 15–18 years of age. Verbal assent was also obtained from study participants ages 15–18 years. Women with severe anemia, as detected by the HemoCue 301, were referred for treatment at the nearest health center immediately, and women with moderate or mild anemia were referred to nutrition centers for enrolment into nutrition and food voucher programs.

### Inclusivity in global research

Additional information regarding the ethical, cultural, and scientific considerations specific to inclusivity in global research is included in (**S2 File**).

## Results

### Sample characteristics

A total of 807 non-pregnant women aged 15–49 consented to participate in the study, and 5 women declined participation. Sample demographic characteristics and average temperature and humidity at the time of sample collection are presented in **Table 1**. Of the surveyed women, 309 women (38%) were currently breastfeeding.

**Table 1:** Descriptive characteristics of non-pregnant women (15–49 y) and environmental conditions in refugee camps in Gambella, Ethiopia^1^.

	Jewi (n = 224)	Kule (n = 222)	Nguennyiel (n = 158)	Pinyudo 1 (n = 203)	Total (n = 807)
**Women’s characteristics**					
Age, y					
15–24 y	91 (40%)	95 (43%)	70 (44%)	63 (31%)	319 (40%)
25–34 y	80 (36%)	78 (35%)	58 (37%)	84 (41%)	300 (37%)
35–44 y	49 (22%)	47 (21%)	27 (17%)	52 (26%)	175 (21%)
45–49 y	4 (2%)	2 (1%)	3 (2%)	4 (2%)	13 (2%)
Currently breastfeeding	68 (30%)	112 (50%)	67 (42%)	62 (31%)	309 (38%)
Breastfeeding a child <6 mo	20 (9%)	50 (23%)	40 (25%)	30 (15%)	140 (17%)
Enrolled in BSFP	12 (5%)	28 (13%)	25 (16%)	18 (9%)	83 (10%)
Mean (SD) weight, kg^2^	52.9 (7.2)	51.1 (6.6)	51.4 (6.2)	52.9 (7.5)	52.0 (7.3)
**Environmental conditions** ^3^					
Mean (SD) temperature, °C	36.4 (1.5)	30.9 (4.5)	30.9 (3.6)	-	32.9 (4.3)
Mean (SD) humidity, %	37.3 (17.0)	50.7 (9.4)	30.5 (9.6)	-	40.3 (15.2)

^1^n (% column), unless noted otherwise.

^2^Weight is not a primary measure but was collected as a proxy for women’s size. Two measurements with a weight <30 kg were removed.

^3^Due to connectivity issues, temperature and humidity were not recorded in Pinyudo 1.

HemoCue 301 measurements were obtained from 800 (99%) women, and 597 (74%) women had both first and second measurements. Rad-67 measurements were obtained from 798 (99%) women, and 798 (99%) women had both measurements. When collecting Rad-67 measurements, departures from protocol occurred for 71 (9%) women for the following reasons: measurement taken on the dominant hand (14, 2%), measurements taken on different hands (16, 2%), low signal stability warning (42, 5%), and other reasons unspecified (2, 0.2%). Of note, 66 of the 71 departures from protocol occurred in Kule camp, which was the first camp in which data were collected. No major differences in demographics of women from whom Hb measurements were taken successfully and women from whom Hb measurements could not be taken were found.

### Accuracy

**Fig 1:**
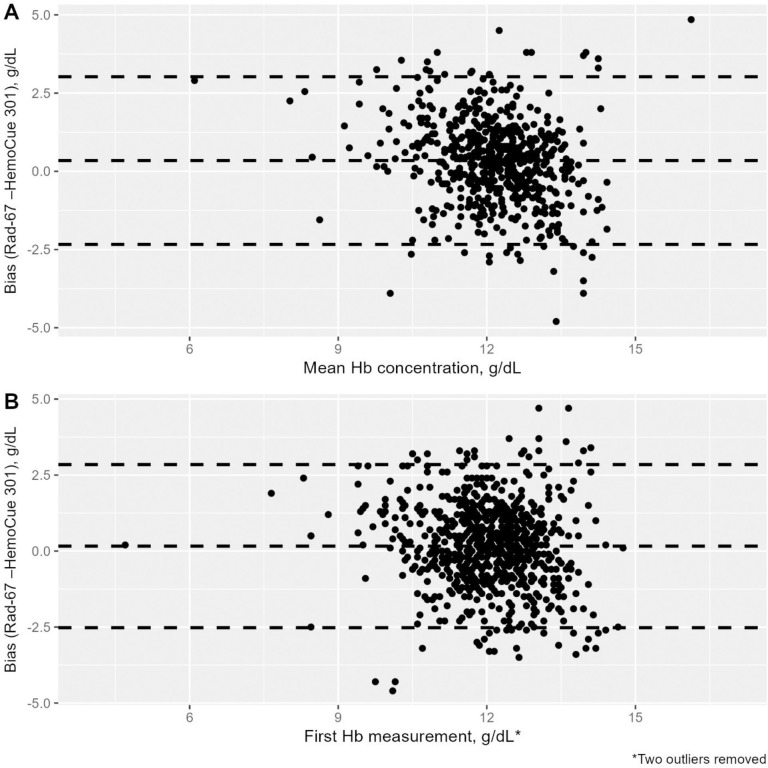
Bland-Altman plots for mean and first hemoglobin (Hb) measurements using the HemoCue 301 and Rad-67. The dashed lines represent the mean difference in measurement between the Rad-67 and HemoCue 301 and 95% limits of agreement.

Average bias of Rad-67 measurements was 1.1 ± 1.0 g/dL when comparing first measurements across devices, using HemoCue 301 as a comparator (**Table 2**). The 95% limit of agreement showed that 95% of differences were within -2.7 to 3.0 g/dL. The Bland-Altman plot, which visualized the accuracy of the Rad-67 **(Fig 1)** indicates that bias was bidirectional, as Rad-67 measurements were not consistently higher or lower than HemoCue 301 measurements. The degree of bias appeared consistent across the x-axis, suggesting a similar bias between NP-WRA regardless of anemia status.

**Table 2:** Bias of hemoglobin measurements from the Rad-67 pulse CO-oximeter as compared to the HemoCue 301^1^.

	Mean bias (SD)^2^	95% LOA^3^	TEM (95% CI)	Relative TEM (%)[24]	Correlation coefficient^4^	P-value
First measurement, g/dL^5^	1.1 (1.0)	-2.7, 3.0	1.0 (-1.0, 3.2)	0.9	0.3	<0.001
Women, Hb <12 g/dL	0.8 (0.7)	-1.8, 2.2	0.8 (-0.7, 2.3)	1.0	0.5	<0.001
Women with two measurements, g/dL^6^	1.1 (0.9)	-2.4, 3.0	1.0 (-0.9, 3.1)	0.9	0.4	<0.001
Women, Hb <12 g/dL	0.9 (0.8)	-1.6, 2.6	0.8 (-0.8, 2.5)	0.9	0.5	<0.001

^1^Hemoglobin, Hb.

^2^Absolute difference, Rad-67 –HemoCue 301.

^3^Limits of agreement are calculated as mean bias ± 1.96 x SD.

^4^Correlation between average Rad-67 and HemoCue 301 measurements and between first Rad-67 and HemoCue 301 measurements.

^5^Comparing the first Rad67 to the first HemoCue 301 measurement.

^6^Women with two Rad67 and two HemoCue 301 measurements.

### Precision

Average Hb concentration measured using the Rad-67 was 12.4 g/dL, compared to 12.0 g/dL using the HemoCue 301 (**Table 3**). The Rad-67 was less internally precise than the HemoCue 301, having an absolute mean difference between first and second measurements of 0.9 g/dL (95% CI 0.8, 0.9), compared to 0.6 g/dL (95% CI 0.5, 0.6) using the HemoCue 301. Technical errors of measurement were 0.9 g/dL for the Rad-67 and 0.6 g/dL for the HemoCue 301. Repeat measurements using the Rad-67 had a positive moderate correlation (r = 0.6, p-value <0.001), though the correlation for repeat measurements using the HemoCue 301 was stronger (r = 0.8, p-value <0.001).

**Table 3:** Precision of repeat hemoglobin measurements from HemoCue 301 and Rad-67 pulse CO-oximeter point-of-care devices^1^.

	HemoCue 301	Rad-67 pulse CO-oximeter
Overall mean (SD) Hb, g/dL^2^	12.0 (1.4)	12.4 (1.2)
Overall mean (SD) perfusion index, %^3^	–	4.5 (3.0)
First measurement, *n*	800	798
Mean (SD) Hb, g/dL	12.0 (1.3)	12.1 (1.2)
Hb range, g/dL	4.6, 16.2	4.8, 24.3
Mean (SD) perfusion index, %	–	4.0 (3.0)
Perfusion index range, %	–	0.2, 20.0
Second measurement, *n*	597	798
Mean (SD) Hb, g/dL	12.0 (1.5)	12.6 (1.2)
Hb range, g/dL	4.1, 16.2	7.9, 19.2
Mean (SD) perfusion index, %	–	5.0 (3.0)
Perfusion index range, %	–	0.0, 20.0
Precision		
Mean difference (SD)^4^	0.6 (0.6)	0.9 (0.8)
95% CI	0.5, 0.6	0.8, 0.9
Technical error of measurement	0.6	0.9
95% limits of agreement	-1.6, 1.6	-2.6, 1.8
Pearson correlation		
Estimate	0.8	0.6
P-value	<0.001	<0.001

^1^Hemoglobin, Hb.

^2^Includes women who did not have a second measurement.

^3^Excludes first (n = 3) and second (n = 5) perfusion index readings outside the valid range (0–20%).

^4^Abs (measurement 1 –measurement 2).

### Regression results

Temperature had a significant negative relationship with bias, using the first Rad-67 and HemoCue 301 measurements (β = -0.03; 95% CI -0.04, -0.01; p-value <0.01) (**Table 4**). Precision of the Rad-67, as measured by mean difference, had a significant negative relationship with age (β = -0.01; 95% CI -0.02, -0.00; p-value = 0.03). Precision was not different by women’s weight, perfusion index, camp, or environmental conditions. There were no statistically significant relationships between precision and bias and women’s weight, age, perfusion index, camp, or environmental conditions using mean measurements where two measurements were available (**S1 Table in S3 File**). Controlling for temperature and humidity, age had a significant, negative correlation with precision (p-value = 0.01); however, the effect size was small (β = -0.01; 95% CI -0.02, -0.00). (**S2 Table in S3 File**).

**Table 4:** Environmental and individual-level factors associated with the precision and bias of the Rad-67 pulse CO-oximeter point-of-care device.

	Precision of the Rad-67			Bias of the Rad-67^1^		
	Coefficient	95% CI	P-value	Coefficient	95% CI	P-value
Univariate analysis						
Temperature	-0.01	-0.03, 0.00	0.07	-0.03	-0.04, -0.01	**<0.01**
Humidity	-0.0	-0.01, 0.00	0.32	-0.00	-0.01, 0.00	0.84
Weight	0.00	-0.01, 0.01	0.48	0.00	-0.01, 0.01	0.41
Age	-0.01	-0.02, -0.00	**0.03**	-0.00	-0.01, 0.01	0.80
Perfusion index	-0.01	-0.03, 0.02	0.55	-0.01	-0.03, 0.01	0.31
Camp^2^						
Kule	0.04	-0.12, 0.20	0.62	0.08	-0.11, 0.26	0.41
Ngunnyiel	0.10	-0.07, 0.27	0.24	0.08	-0.12, 0.28	0.43
Pinyudo I^3^	0.04	-0.12, 0.20	0.65	-0.08	-0.27, 0.11	0.40

^1^Calculated using first Rad-67, perfusion index, and HemoCue 301 measurements.

^2^Camp is a fixed effect. Jewi is the reference camp.

^3^HemoCue 301 measurements were not taken.

### Classification of anemia

Total anemia (Hb concentration <12.0 g/dL) was detected in 39% of women as classified by the Rad-67 compared with 47% of women as classified by the HemoCue 301 using the first measurement (**Table 5**). The prevalence of mild, moderate, and severe anemia was 23%, 13%, and <1%, respectively, as evaluated by the Rad-67.

**Table 5:** Proportion of non-pregnant women (15–49 y) with mild, moderate, and severe anemia as classified by hemoglobin concentrations from HemoCue 301 and Rad-67 pulse CO-oximeter point-of-care devices^1^,^2^.

	HemoCue 301	Rad-67 pulse CO-oximeter	
**First measurement**			
Total anemia, Hb <12.0 g/dL	379 (47%)	312 (39%)	
Mild, Hb 11.0–11.9 g/dL	236 (30%)	204 (26%)	
Moderate, Hb 8.0–10.9 g/dL	140 (18%)	104 (13%)	
Severe, Hb <8.0 g/dL	3 (<1%)	4 (<1%)	
**Women with two measurements**			
Total anemia, Hb <12.0 g/dL	282 (47%)	261 (33%)	
Mild, Hb 11.0–11.9 g/dL	168 (28%)	192 (24%)	
Moderate, Hb 8.0–10.9 g/dL	111 (19%)	66 (8%)	
Severe, Hb <8.0 g/dL	3 (<1%)	3 (<1%)	

^1^ Hemoglobin, Hb.

^2^Values are n (%).

The Rad-67 correctly classified total anemia in 60% of women, using HemoCue 301 as a comparator (**Table 6**). The Rad-67 had 49% sensitivity and 70% specificity for detecting total anemia, compared with the HemoCue 301. Sensitivity did not increase for detecting mild (28%), moderate (31%), and severe (33%) anemia; however, specificity did improve for detecting mild (75%), moderate (91%), and severe (99%) anemia. Results were similar using mean measurements when two measurements were available.

**Table 6:** Sensitivity, specificity, and correct classification rates for the Rad-67 pulse CO-oximeter device using a cut-off of 12.0 g/dL^1^.

	Sensitivity	Specificity	Correct classification
Total anemia, first Rad-67 measurement only	49	70	60
Mild anemia	28	75	61
Moderate anemia	31	91	80
Severe anemia	33	99	99
Total anemia, average of both Rad-67 measurements^2^	45	76	61

^1^Values are %.

^2^Women with two HemoCue 301 measurements.

### Focus group discussions

A total of 23 supervisors and 15 enumerators participated in focus group discussions. Supervisors and enumerators identified several issues regarding data collection that may have affected (or were perceived to affect) Rad-67 performance. The following section highlights key themes of responses related to (1) stability; (2) participant perception and experience; and (3) recommendations for future use.

#### Stability

Average temperature and humidity at the time of sampling were 32.9°C and 40.3%, respectively. Enumerators and supervisors highlighted weather as a barrier to taking measurements and reported length of measurement as dependent on temperature:

*When temperatures rise or get heated, Rad-67 or Masimo needs stability, as one of my pals mentioned. The drawback is that it is susceptible to environmental factors like humidity and temperature.* (Supervisor)

*The disadvantages are when there is a windy or warm environment (such as the sun)*, *Masimo or Rad-67 have problems with their displays*, *signal perfusion*, *stability*, *and battery life*. (Enumerator)

*The difficulty with the Masimo machine is that the results differ in range when compared to HemoCue 301*, *and at low temperatures*, *there is no signal*. (Supervisor)

An additional challenge noted was movement by the participant before and during measurements as a limiting factor to taking reliable repeat measurements. Issues with participants moving during device reading caused measurements to take longer than anticipated. Eleven enumerators and seven supervisors participating in focus groups reported concerns regarding device stability during participant movement:

*It [Rad-67] won’t be suitable for site work because the machine is too sensitive. The machine’s output will fluctuate when there is movement. The device is not appropriate for[refugee] sites in my opinion, because it requires a stable environment, such as a hospital. The machine cannot be used for this type of survey since there is movement, which will cause it to display a different result.* (Supervisor)

*I therefore advise the Hb machine here because the Masimo machine requires a stable space and takes longer to measure (more than 10 minutes)*, *therefore*, *we cannot have stable area at the community level*. (Supervisor)

*The machine [Rad-67] has slight disadvantages*, *like it’s not effective for patients because it needs stability*. *Most of the patients are restless*, *so it’s not suitable for patients*. (Enumerator)

#### Perceived participant perception and experience

Prior to the inception of this research, community sensitization was organized with local leaders in each camp to communicate research aims, discuss plans for disseminating findings among the local population, and respond to any concerns. Despite these activities, some participants had reservations regarding taking measurements using the Rad-67:

*The participant, nevertheless, believed that their blood was being pulled covertly because the machine was new; however, after we gave them informed consent, they accepted the machine.* (Supervisor)

*At first*, *they had some reservations about the device and had questions*, *but after being shown how it worked and seeing that no finger was pierced*, *they decided to accept it*. (Supervisor)

*There were no difficulties; however*, *nobody in the community is aware of the machine*. *The majority of the residents of the community believed that we would be taking blood samples from them; thus*, *I propose that before taking measurements*, *we use a community mobilizer to raise awareness among the residents*. (Supervisor)

Despite these challenges, survey enumerators and supervisors perceived that women were overwhelmingly in favor of using the Rad-67 as a non-invasive means of measuring Hb concentration:

*Because there is no piercing, the participants’ dread is reduced and they experience no terror, which has many benefits.* (Supervisor)

*Most of the people in this refugee camp do not want to give blood samples*. *They readily accept the measurement after they realize we did not draw their blood*. (Supervisor)

*Regarding acceptability*, *initially*, *it was challenging but through time they observed the procedure is non-invasive*, *so they accept the Masimo machine…When we compare with HemoCue*, *the Rad-67 is more acceptable because the community perceives the loss of blood when they observe bleeding from the beneficiary*. (Enumerator)

#### Recommendations for future use from focus group participants

Overall, the Rad-67 received favorable feedback from supervisors and enumerators:

*I will advise UNHCR to use the device, but we need to conduct a thorough assessment of its drawbacks and how well it performs in various weather conditions.* (Supervisor)

*In the future*, *Rad-67 is advised if the machine’s limitations are resolved and it outperforms HemoCue 301*. (Supervisor)

## Discussion

This study evaluated the accuracy and precision of Hb measurements among non-pregnant women aged 15–49 years in Gambella Refugee Camps using the Rad-67, as compared to the HemoCue 301. A non-invasive alternative to the HemoCue 301 would allow for increasing sample size in SENS, expanding screening of non-severe cases that have not yet presented with signs and symptoms, and potentially more frequent assessments of anemia in refugee camp settings. In addition to programmatic benefits, a non-invasive alternative could decrease marginal costs, lessen the burden of supply chain challenges when procuring commodities for sample collection, and eliminate infection prevention control needs associated with the HemoCue 301. To our knowledge, this study is among the first to evaluate the validity and utility of pulse CO-oximetry in a refugee camp setting. It thus provides useful information on the real-world performance of this tool in this setting.

On average, the Rad-67 overestimated Hb (0.4 g/dL) and underestimated the population prevalence of anemia (39% compared to 47%), as compared to the HemoCue 301. However, the estimated prevalence of anemia via the Rad-67 is consistent with the 33.9% prevalence of anemia in NP-WRA observed during the 2021 SENS survey in Gambella (SENS 2021). The HemoCue 301 and Masimo pulse CO-oximeters have been found to both under- and overestimate Hb concentrations. Our findings of HemoCue’s lower Hb measurements and Rad-67’s overestimation of Hb is in contrast with previous studies. Whitehead et al. conducted a systematic review of methods used to measure Hb in clinical and field settings and found that when compared to the automated hematology analyzer, the HemoCue 301 reported higher Hb concentrations (0.5–6.0 g/L) in capillary blood, and non-invasive Masimo devices under- and overestimated Hb concentrations (+/-0.3–14.0 g/L) [25]. A study in pregnant women compared the Masimo Rainbow SET^®^ Radical-7™ pulse CO-oximeter to the HemoCue Hb 201+ and found that the CO-oximeter gave lower Hb readings and was less accurate than the HemoCue Hb 201+ capillary blood measurements when compared with laboratory measurements [26]. Although existing studies included different populations (i.e., men, pregnant women, children, stable populations), settings, and/or versions of HemoCue and pulse CO-oximeter devices, we found similar findings of lesser accuracy and precision among Rad-67 readings.

Bias of Rad-67 measurements (1.1 g/dL) was much higher than what was observed in the manufacturer’s accuracy data (0.1 g/dL) [18] and previous studies comparing the Rad-67 to a laboratory reference [14,15]. Additionally, our observed correlation between Rad-67 and HemoCue 301 measurements was lower (r = 0.3–0.4) than observed when the Rad-67 was compared to a laboratory reference (r = 0.812) [15]. We did not compare Rad-67 measurements to a gold standard (lab reference) and therefore do not have a conventional measure of bias. It is plausible that the higher bias observed is due to our comparison to the HemoCue 301 rather than to a standard reference. In a study comparing biases between the non-invasive Pronto-7 and HemoCue 201+ to a standard reference, the HemoCue 201+ measurement was, on average, 0.98 g/dL lower than the laboratory value [27]. Thus, our comparison to the HemoCue 301, known to both over- and underestimate Hb concentration, could explain the high bias observed. The Bland-Altman plots illustrate that bias is similar between women with moderate and severe anemia. However, since there is variation in the direction of bias, a correction factor cannot be applied to this data. Bias did not improve with a second Rad-67 reading, suggesting that one reading may be sufficient, which could help the feasibility of enumerators taking measurements.

In comparison to the HemoCue 301, the Rad-67 was less precise (0.9 g/dL compared to 0.6 g/dL). Lower precision and wider limits of agreement of Masimo pulse CO-oximeters, in comparison to HemoCue 301 devices, have been reported previously [28]. However, in our refugee setting, Rad-67 precision is in agreement with optimal published performance (1.0 g/dL) [18]. This limitation and the meaningfulness of a 1.0 g/dL difference in repeat measurements to anemia classification should be considered before basing clinical decisions on Rad-67 measurement alone.

The Rad-67 had a sensitivity of 49%, using HemoCue 301 as the comparator. This finding is consistent with Young et. Al [29]. who assessed anemia in refugees (women, men, and children) arriving in Atlanta, GA and observed 45.7% sensitivity using the Masimo Pronto compared to the laboratory reference. Specificity was excellent in participants having moderate and severe anemia, which suggests the Rad-67 could be used as an initial screening test that could likely rule out those without moderate and severe anemia, and those with anemia could then be sent for follow-up testing via the HemoCue 301. Correct classification is in line with what has been observed previously in a study comparing a Masimo non-invasive device to a standard method [19] and further displays the need for additional testing after Rad-67 screenings.

Focus group discussions provided insight into Rad-67 operations in the field that could be improved upon to provide more reliable measurements during future assessments. Improvements in training field staff are needed for future implementation in refugee settings, such as increasing the length of time for training, understanding, and troubleshooting device display errors, and counseling on techniques for preventing the motion of participants during measurements. Most departures from protocol occurred in the first camp where data was collected, highlighting the importance of improved and ongoing training. Training materials could be updated to include graphics and written instructions in local languages, with standard protocols for troubleshooting common errors in the field. A basic overview of biologically plausible Hb concentration range and WHO cut-offs for anemia could facilitate a better interpretation of measurements in the field and provide a logical series of next steps (i.e., take a repeat measurement; refer to the clinic for follow-up measurement via the HemoCue 301) based on readings. Further sensitization in the camps is needed to reinforce the purpose of the Rad-67 and how Hb measurements are taken without piercing the skin.

The use of the Rad-67 in a refugee setting highlighted several challenges that may have contributed to the low classification of anemia and less precision compared to the widely implemented HemoCue 301 device. Stability of the Rad-67 in varied environmental conditions was a major concern expressed by enumerators and supervisors. Though Rad-67 specifications state an operating temperature of up to 35°C and operating humidity of up to 95% [18], users observed issues with functionality in locally typical environmental conditions during use. The Rad-67 manual states that total Hb accuracy has not been validated with movement [18]. Motion during a measurement using pulse oximeters, as discussed during FGDs, can cause inaccurate Hb readings [30]. Difficulty taking measurements during participant motion, which can result in taking a longer time to complete measurements, when using a Masimo non-invasive device has been noted previously [29]. The potential effect of participant movement before and during measurement on precision requires further investigation.

Given the low sensitivity of the Rad-67 for identification of anemia in comparison with cases identified by the HemoCue 301 in our study, widespread replacement of the HemoCue 301 by the Rad-67 for the purpose of anemia detection in Gambella refugee camps is not recommended. Individual anemia cases among women of reproductive age may be missed if solely using the Rad-67 for diagnostic screening. A potential underestimation of population estimates of anemia may have programmatic and funding implications. However, Rad-67 could be used in this setting in a more nuanced way. With its very high specificity for moderate and severe anemia, it could be used as an initial screening test with subsequent confirmation with HemoCue 301. It could also be used as a monitoring tool for WRA already identified with anemia to track changes while receiving treatment. This suggestion aligns with that of a study comparing a Masimo pulse CO-oximeter to a standard laboratory test that found unacceptable precision, acceptable accuracy, and high sensitivity, and suggested using the pulse CO-oximeter as a screening tool with subsequent confirmation using standard techniques [31].

### Limitations

This study is subject to several limitations. First, this study did not compare Rad-67 results to a gold standard, such as the automated hemoglobin analyzer. Availability of functional automated analyzers and adequate reagents in the region impacted the ability to have a laboratory reference comparison. Additionally, there were logistical and ethical concerns about taking venous blood samples in the refugee shelters. Therefore, the interpretation of anemia classification rates, accuracy, and bias of the Rad-67 is limited to a comparison of measurements of capillary blood samples which has its own limitations [25,32,33]. Second, study results cannot be extrapolated to anemia measurement in children, pregnant women, or men, as our study did not include these populations. Accuracy and precision of HemoCue 301 and Masimo devices in children have been evaluated previously in low- or middle-income settings [19,34]. However, the Rad-67 is only indicated for the measurement of total Hb concentration in adults. Third, our study is powered to draw conclusions across camps. Therefore, camp-specific variation in measurement could not be investigated statistically to determine intra-camp errors in measurement. Finally, while enumerators and supervisors provided feedback on the perceived acceptability of participants having measurements taken using the Rad-67, participants were not directly asked about their experience. Therefore, we cannot conclude that the Rad-67 was accepted by all participants.

## Conclusions

Previous assessments provided evidence that pulse CO-oximetry could provide a highly accurate and precise measurement of anemia. This study evaluated whether these findings could be replicated in a refugee camp setting, being less controlled than in a clinical setting and having unfavorable environmental conditions. However, our results suggest that Rad-67 measurements were not of acceptable precision or sensitivity for widespread adoption for the detection of anemia among this population. Additional training, testing, and validation, using a standard reference as a comparator, in refugee contexts are needed before the Rad-67 can be recommended as a stand-alone diagnostic test in future SENS. Use of non-invasive measurement of Hb has the potential to help address WHO’s call for accelerated action to reduce anemia in women of reproductive age [4,35]. Tradeoffs between higher acceptability and lower cost of the Rad-67 should be considered against the invasiveness, but higher reliability of the HemoCue 301 in addition to logistical and programmatic considerations.

## Supporting information

S1 File Focus group discussion guides. (PDF)

S2 File Inclusivity in global research. (PDF)

S3 File Supporting S1 and S2 Tables. (PDF)
