# Supplementary material for: Comparison of a non-invasive point-of-care measurement of anemia to conventionally used HemoCue devices in Gambella refugee camp, Ethiopia, 2022
Source: PLoS One. 2025 Jan 13;20(1):e0313319. doi: 10.1371/journal.pone.0313319 (PMC11729968; doi:10.1371/journal.pone.0313319)
Supplement: S1 File — (PDF) [file pone.0313319.s001.pdf]

Focus Group Discussion Guides

Supervisors

Selecting focus group participants: one focus group should include supervisors (representation from each of the six teams) of enumerators who conducted evaluation measurements. Informed consent will be obtained from all focus group participants.

Selecting focus group facilitators: all focus groups will be conducted by a team of three researchers including the research investigators (acting as moderators), notetaker and logistics coordinator.

**BACKGROUND INFORMATION**	
FGD date<br>FGD moderator name<br>Note taker name<br>Logistics coordinator name<br>Location – Camp	
FGD GROUP	☐ Supervisor ☐ Enumerator
**CONSENT**	
[INSERT APPROVED CONSENT STATEMENT]	

PARTICIPANT INFORMATION

In which camp(s) were you survey supervisor?	Participant		Team Information	Consent to participate
	Age	Sex	Team #(s)	
				Yes ☐ No ☐
				Yes ☐ No ☐
				Yes ☐ No ☐

1. Your experience with training on the Masimo and HemoCue

READ: We are interested in your experiences with training on how to use the Masimo Rad-67 [which I will refer to as Masimo] and HemoCue 301+ [which I will refer to as HemoCue].

1.1 What is your perception of the training for the Masimo? How could training on the Masimo be improved?
   - Probe: Was the length of training appropriate? Were there additional topics that should have been covered? Did training fully prepare you for use in the field?

1.2 What is your perception of the training for the HemoCue? How could training on the HemoCue be improved?
   - Probe: Was the length of training appropriate? Were there additional topics that should have been covered? Did training fully prepare you for use in the field?

2. Your experience using the Masimo and HemoCue

READ: We are also interested in your experiences using the Masimo and HemoCue in the field. The next set of questions will focus on the Masimo device.

2.1 Can you tell us what you like about using the Masimo? What are its greatest attributes?

- Probe: Was it easy to use? Was it easy to troubleshoot any issues the enumerators may have experienced?

2.2 Is there anything you did not like about using the Masimo? What were your greatest challenges?

- Probe: Were there any technical glitches? Did it ever stop working?

2.3 What was the perception of women with respect to using the Masimo? What was the perception of the younger participants (15-17 yrs. old)?

- Probe: Was it well accepted? Did you get a sense of if it was preferred to the HemoCue?

READ: Now, I will ask about your experience using the HemoCue device.

2.4 Can you tell us what you like about using the HemoCue? What are its greatest attributes?

- Probe: Was it easy to use? Was it easy to troubleshoot any issues the enumerators may have experienced?

2.5 Is there anything you did not like about using the HemoCue? What were your greatest challenges?

- Probe: Were there any technical glitches? Did it ever stop working?

2.6 What was the perception of women with respect to using the HemoCue? What was the perception of the younger participants (15-17 yrs. old)?

- Probe: Was it well accepted? Did you get a sense of if it was preferred to the Masimo?

3. Barriers to Measurement

READ: Now we will ask about barriers to measurement.

3.1 From the data we collected, we saw that measurements were frequently taken on the dominant hand or a different finger. Based on what you observed, why do you think there were departures from protocol?

- Probe: Are there measures the study team could have put in place to prevent departures from protocol? In the future, are there any additional measures you would recommend?

3.2 Now that you have supervised enumerators taking measurements, do you feel comfortable using the Masimo? Rad67?

- Probe: Do you feel comfortable training new users?

4. Considerations for future use of the devices

READ: Lastly, we would like to hear about any considerations you may have should UNHCR use these devices in the future.

4.1 Based on your experience, what considerations should UNHCR have in deciding on using these devices for future SENS surveys in Ethiopia or other refugee settings?

- Probe: Do you have any thoughts on whether the Masimo or HemoCue is better suited for refugee settings?

4.2 Based on your experience, what considerations should UNHCR have in deciding on using these devices in other settings (health facilities, house to house screenings)?

- Probe: Do you have any thoughts on whether the Masimo or HemoCue is better suited for other settings?

5. Final remarks

5.1 This is the end of the discussion. Would you like to share any additional thoughts?

Enumerators

Selecting focus group participants: three focus groups should include survey enumerators (representation from each of the six teams) who conducted evaluation measurements. Each group will have approximately 5-7 people. Written informed consent will be obtained from all focus group participants.

Selecting focus group facilitators: all focus groups will be conducted by a team of three researchers including the research investigators (acting as moderators), notetaker and logistics coordinator.

**BACKGROUND INFORMATION**	
FGD date<br>FGD moderator name<br>Note taker name<br>Logistics coordinator name<br>Location – Camp	
FGD GROUP	☐ Supervisor ☐ Enumerator
**CONSENT**	
[INSERT APPROVED CONSENT STATEMENT]	

PARTICIPANT INFORMATION

**Role during survey (team lead/ anthro measure / Hb measure)**	**Participant**		**Team Information**	**Consent to participate**
	**Age**	**Sex**	**Team #(s)**	
				Yes ☐ No ☐
				Yes ☐ No ☐
				Yes ☐ No ☐

1. Your experience with training on the Masimo and HemoCue

READ: We are interested in your experiences with training on how to use the Masimo Rad-67 [which I will refer to as Masimo] and HemoCue 301+ [which I will refer to as HemoCue].

1.1 What is your perception of the training for the Masimo? How could training on the Masimo be improved?
- Probe: Was the length of training appropriate? Were there additional topics that should have been covered? Did training fully prepare you for use in the field?

1.2 What is your perception of the training for the HemoCue? How could training on the HemoCue be improved?
- Probe: Was the length of training appropriate? Were there additional topics that should have been covered? Did training fully prepare you for use in the field?

2. Your experience using the Masimo and HemoCue

READ: We are also interested in your experiences using the Masimo and HemoCue in the field. The next set of questions will focus on the Masimo device.

2.1 Can you tell us what you like about using the Masimo? What are its greatest attributes?

- Probe: Was it easy to use?

2.2 Is there anything you did not like about using the Masimo? What were your greatest challenges?

- Probe: Were there any technical glitches? Did it ever stop working?

2.3 What was the perception of women with respect to using the Masimo? What was the perception of the younger participants?

- Probe: Was it well accepted? Did you get a sense of if it was preferred to the HemoCue?

READ: Now, I will ask about your experience using the HemoCue device.

2.4 Can you tell us what you like about using the HemoCue? What are its greatest attributes?

- Probe: Was it easy to use?

2.5 Is there anything you did not like about using the HemoCue? What were your greatest challenges?

- Probe: Were there any technical glitches? Did it ever stop working?

2.6 What was the perception of women with respect to using the HemoCue? What was the perception of the younger participants (15-17 yrs. old)?

- Probe: Was it well accepted? Did you get a sense of if it was preferred to the Masimo?

3. Barriers to Measurement

READ: Now we will ask about barriers to measurement.

3.1 From the data we collected, we saw that when piloting the measurements, measurements were frequently taken on the dominant hand or a different finger. Based on your experience, why do you think there were departures from protocol?

- Probe: Are there measures the study team could have put in place to prevent departures from protocol? In the future, are there any additional measures you would recommend?

3.2 Now that you have experience taking measurements with these devices, do you feel comfortable using the Masimo? Rad67?

- Probe: Would you need additional training if you were asked to take measurements again?

4. Considerations for future use of the devices

READ: Lastly, we would like to hear about any considerations you may have should UNHCR use these devices in the future.

4.1 Based on your experience, what considerations should UNHCR have in deciding on using these devices for future SENS surveys in Ethiopia or other refugee settings?

- Probe: Do you have any thoughts on whether the Masimo or HemoCue is better suited for refugee settings?

4.2 Based on your experience, what considerations should UNHCR have in deciding on using these devices in other settings (health facilities, house to house screenings)?

- Probe: Do you have any thoughts on whether the Masimo or HemoCue is better suited for other settings?

5. Final remarks

5.1 This is the end of the discussion. Would you like to share any additional thoughts?
