# Supplementary material for: Comparison of a non-invasive point-of-care measurement of anemia to conventionally used HemoCue devices in Gambella refugee camp, Ethiopia, 2022
Source: PLoS One. 2025 Jan 13;20(1):e0313319. doi: 10.1371/journal.pone.0313319 (PMC11729968; doi:10.1371/journal.pone.0313319)
Supplement: S3 File — (PDF) [file pone.0313319.s003.pdf]

Supporting Tables S1 and S2

Table S1. Environmental and individual-level factors associated with bias of the Rad-67 pulse CO-oximeter point-of-care device, using averaged repeat measurements

	Bias of the Rad-67		
	Coefficient	95% CI	P-value
	Univariate analysis		
Temperature	-0.01	-0.02, 0.01	0.53
Humidity	-0.00	-0.01, 0.00	0.15
Weight	0.00	-0.01, 0.01	0.97
Age	0.00	-0.01, 0.01	0.93
Perfusion index	0.00	-0.02, 0.03	0.97
Camp[1]			
Kule	-0.06	-0.23, 0.10	0.46
Nguennyiel	0.09	-0.10, 0.26	0.36
Pinyudo I[2]	-	-	-

[1]Camp is a fixed effect. Jewi is the reference camp.

[2]Second measurements were not taken using the HemoCue 301.

Table S2. Environmental and individual-level factors associated with the precision and bias of the Rad-67 pulse CO-oximeter point-of-care device, using temperature and humidity as explanatory variables

	**Precision of the Rad-67**			**Bias of the Rad-67**[1]			**Bias of the Rad-67**[2]		
	Coefficient	95% CI	P-value	Coefficient	95% CI	P-value	Coefficient	95% CI	P-value
Temperature + humidity									
Temperature	-0.02	-0.04, -0.00	**0.03**	-0.02	-0.04, -0.00	**0.02**	-0.01	-0.02, 0.01	0.45
Humidity	-0.00	-0.01, 0.00	0.19	-0.00	-0.01, 0.00	0.50	-0.00	-0.01, 0.00	0.12
Temperature + humidity + weight									
Temperature	-0.02	-0.04, -0.00	**0.02**	-0.02	-0.04, -0.00	**0.02**	-0.01	-0.02, 0.01	0.44
Humidity	-0.00	-0.01, 0.00	0.18	-0.00	-0.01, 0.00	0.48	-0.00	-0.01, 0.00	0.12
Weight	0.00	-0.01, 0.02	0.29	0.00	-0.01, 0.01	0.51	0.00	-0.01, 0.01	0.75
Temperature + humidity + age									
Temperature	-0.02	-0.04, -0.00	**0.04**	-0.02	-0.04, -0.00	**0.02**	-0.01	-0.02, 0.01	0.45
Humidity	-0.00	-0.01, 0.00	0.20	-0.00	-0.01, 0.00	0.50	-0.00	-0.01, 0.00	0.12
Age	-0.01	-0.02, -0.00	**0.01**	0.00	-0.01, 0.01	0.88	0.00	-0.01, 0.01	0.95
Temperature + humidity + perfusion index									
Temperature	-0.02	-0.03, -0.00	**0.04**	-0.02	-0.04, -0.00	**0.02**	-0.01	-0.02, 0.01	0.48
Humidity	-0.00	-0.01, 0.00	0.22	-0.00	-0.01, 0.00	0.57	-0.00	-0.01, 0.00	0.16
Perfusion index	-0.01	-0.04, 0.01	0.33	-0.02	-0.04, 0.01	0.21	0.00	-0.02, 0.03	0.95
Temperature + humidity + camp[3]									
Temperature	-0.02	-0.05, -0.00	**0.03**	-0.03	-0.05, -0.00	**0.04**	-0.01	-0.03, 0.01	0.46
Humidity	-0.00	-0.01, 0.00	0.42	-0.00	-0.01, 0.00	0.48	-0.00	-0.01, 0.00	0.44
Kule	-0.10	-0.32, 0.13	0.42	-0.03	-0.29, 0.21	0.77	-0.07	-0.30, 0.15	0.52
Ngunnyiel	-0.04	-0.26, 0.18	0.71	-0.08	-0.32, 0.17	0.52	0.04	-0.19, 0.26	0.75
Pinyudo I[4]	-	-	-	-	-	-	-	-	-

[1]Calculated using first measurements
[2]Calculated using averaged repeat measurements
[3]Camp is a fixed effect. Jewi is the reference camp.
[4]Second HemoCue 301 measurements were not taken and temperature and humidity were not measured.
